# Plasma lutein concentrations are related to dietary intake, but unrelated to dietary saturated fat or cognition in young children

**DOI:** 10.1017/jns.2014.10

**Published:** 2014-05-07

**Authors:** Kelly A. Mulder, Sheila M. Innis, Betina F. Rasmussen, Brian T. Wu, Kelly J. Richardson, David Hasman

**Affiliations:** Nutrition and Metabolism Program, Child and Family Research Institute, Department of Paediatrics, Faculty of Medicine, University of British Columbia, Vancouver, BC, Canada V5Z 4H4

**Keywords:** Lutein, Children, Dietary fat, Saturated fat, Cognition, IQ, intelligence quotient, KABC-II, Kaufman Assessment Battery, PPVT, Peabody Picture Vocabulary Test

## Abstract

Lutein and zeaxanthin are xanthophyll carotenoids present in highly pigmented vegetables and fruits. Lutein is selectively accumulated in the brain relative to other carotenoids. Recent evidence has linked lutein to cognition in older adults, but little is known about lutein in young children, despite structural brain development. We determined lutein intake using FFQ, one 24 h recall and three 24 h recalls, plasma lutein concentrations and their association with cognition in 160 children 5·6–5·9 years of age, at low risk for neurodevelopmental delay. Plasma lutein was skewed, with a median of 0·23 (2·5th to 95th percentile range 0·11–0·53) µmol/l. Plasma lutein showed a higher correlation with lutein intake estimated as the average of three 24 h recalls (*r* 0·479; *P* = 0·001), rather than one 24 h recall (*r* 0·242; *P* = 0·003) or FFQ (*r* 0·316; *P* = 0·001). The median lutein intake was 697 (2·5th to 95th percentile range 178–5287) µg/d based on three 24 h recalls. Lutein intake was inversely associated with SFA intake, but dietary fat or SFA intakes were not associated with plasma lutein. No associations were found between plasma lutein or lutein intake and any measure of cognition. While subtle independent effects of lutein on child cognition are possible, separating these effects from covariates making an impact on both child diet and cognition may be difficult.

Carotenoids are naturally occurring fat-soluble pigments synthesised by plant, but not mammalian, cells. They are responsible for the red, orange and yellow pigments in some fruits and vegetables. While more than 650 naturally occurring carotenoids have been characterised, only about fifty have been identified in human tissues, among which β-carotene is probably the best known^(^^1^^)^. Lutein and zeaxanthin are xanthophyll (oxygenated) carotenoids known to cross the blood–brain barrier and preferentially accumulate in the macular region of the retina. Lutein, but not zeaxanthin, also shows preferential accumulation in the brain^(^^2^^,^^3^^)^. Lutein and zeaxanthin are isomers that differ in the position of one double bond. Their functions in plants stem from their long conjugated chain of double bonds, which has light-absorbing properties and is highly susceptible to oxidative degradation. To date, the roles of lutein and zeaxanthin in human tissues are best understood for the retina where they appear to function in protecting from blue light damage and oxidative stress, possibly conferring protection against diseases such as age-related macular degeneration^(^^4^^–^^9^^)^.

Recently, attention has been drawn to the selective accumulation of lutein relative to other carotenoids in the brain, as well as the transfer of lutein across the placenta^(^^2^^,^^10^^)^. Analyses of autopsy tissues have shown that these xanthophylls account for 66–77 % of all carotenoids in the human brain^(^^2^^)^, with studies in non-human primates demonstrating significant correlations between lutein and zeaxanthin concentrations in the macula and brain^(^^3^^)^. While information on the role of lutein in the brain is limited, improved verbal fluency, memory and learning were reported following lutein supplementation of older adults^(^^11^^)^, and serum lutein and postmortem measures of brain lutein were significantly related to better cognition in octogenarians^(^^12^^)^. With the exception of some studies on lutein bioavailability in infants^(^^13^^)^, little is known about lutein in early childhood although brain synapse accumulation continues to peak at 5–6 years of age^(^^14^^,^^15^^)^.

Although it is clear that all human plasma and tissue lutein must originate from the diet, current information relating lutein intake to plasma lutein in adults varies from non-significant to highly significant correlations^(^^16^^–^^21^^)^, with no correlations in children^(^^22^^,^^23^^)^. Other studies show high inter-individual variability in plasma or serum lutein in response to intake, not explained by differences in plasma TAG or cholesterol^(^^24^^–^^26^^)^. While differences in absorption or bioavailability have been suggested, the lipophilic water-insoluble nature of xanthophylls suggests that fat intake may also influence lutein absorption and thus status. In this regard, recent experimental studies have suggested that diets rich in SFA result in higher bioavailability of lutein than diets rich in MUFA and PUFA^(^^27^^)^. However, information on whether dietary fat is associated with lutein status in humans following their usual diets is limited, and nothing is known for children. In the present study, we determined lutein intake and the effect of SFA intake on plasma lutein, and addressed the possible influence of lutein status on cognition in 160 children 5·75 years of age. Because foods rich in lutein, such as dark green vegetables and egg yolk, may not be eaten daily we also assessed the importance of dietary methodology reflecting both short- and longer-term intakes for determining the association between lutein intake and lutein status.

## Subjects and methods

### Subjects and design

This was a cross-sectional study of healthy children aged 5 years 9 months, with no known health problems, living in Vancouver, Canada. Children were recruited from the community and enrolled together with a parent or legal guardian. Because the present study involved assessment of neurodevelopment, children born preterm (<37 weeks gestation), with congenital or acquired disease, or who had severe food allergies or immune disorders likely to make an impact on growth and development were ineligible. On enrolment, each child was assigned a unique, computer-generated, random code held in sealed opaque envelopes and this code was used on all data collection forms and blood samples. The present study was conducted according to guidelines laid down in the Declaration of Helsinki and all procedures involving human subjects were approved by the Committee for Ethical Review of Research Involving Human Subjects at the University of British Columbia and the British Columbia Children's and Women's Hospital. Written informed consent was obtained from a parent or legal guardian for each child before enrolment.

### Subject characteristics and dietary assessments

Sociodemographic data, including maternal age, highest level of education attained, ethnic background, household income and family size, were collected by questionnaire. Because of the limitations of using income and formal education as a proxy for the mother's intelligence quotient (IQ), we assessed each mother's IQ using the Test of Nonverbal Intelligence-3 (TONI-3), which assesses aptitude, abstract reasoning and problem solving^(^^28^^)^. Each child's weight and height was measured in light clothing without shoes, then *Z*-scores for weight-for-age, height-for-age and BMI-for-age were calculated using the WHO Anthroplus anthropometric calculator software (version 1.0.4). Dietary intake was assessed using a FFQ that gathered information on the foods eaten, frequency of intake and portion sizes over the previous month, and using 24 h recalls. The FFQ was designed to record the frequency and amounts of all foods and beverages typically consumed by children in our population. At the end of each food category, for example vegetables, an open-ended question asked if any other vegetables were eaten, and type, quantity and frequency were recorded. The FFQ and one 24 h recall record of all food and beverages consumed on the day before blood collection were completed by in-person interview with the parent or legal guardian, using food models and measuring utensils. Two further 24 h recall records were collected by telephone at random over 14 d, all using a standardised five-pass technique identical to that used in the in-person 24 h record^(^^29^^)^. The dietary information was analysed using nutrient analysis software with a Canadian food nutrient database and the US Department of Agriculture database on the lutein (sum of lutein + zeaxanthin) content of foods (ESHA Food Processor SQL, version 10.10.0.0; ESHA Research).

### Blood collection and plasma analysis

Fasting venous blood was collected using EDTA as the anticoagulant, the plasma separated by centrifugation, sampled and stored at –80°C until analysed. Lutein was analysed by HPLC (Waters Alliance 2695; Milford) equipped with a photodiode array detector (Waters) based on the method of Yeum *et al.*^(^^24^^)^ and Hess *et al.*^(^^30^^)^ with slight modification. Briefly, separation was achieved on a C18 column, 150 mm × 2·1 mm, 3 µm particle size (Agilent Technologies). The mobile phases were 1 mm-ammonium acetate in water (A) and 1 mm-ammonium acetate in methanol (B) commencing at 6 % A:94 % B for 10 min, followed by 100 % B for 5 min, returning to 6 % A:94 % B to re-equilibrate the column before the next analysis. For practicality, lutein + zeaxanthin were eluted as a single peak with an analysis time of <10 min, using Tocol (MJS BioLynz, Matreya #1797) as the internal standard, and are referred to as lutein. The lower limit of quantification was 0·26 pmol and the CV for sample analysis was <7 %.

### Child developmental assessments

Child development was assessed using the Kaufman Assessment Battery (KABC-II), which is designed as a test of IQ for children 2·5 to 12 years of age^(^^31^^)^. The test involves a sequential processing scale and simultaneous processing scale, with scores from parts of these two scales used to give a mental composite scale and learning ability scores. The sequential processing scale measures a child's ability to solve problems requiring ordered arrangement of stimuli while the simultaneous processing scale requires the child to solve organisational/spatial problems that require arrangement of multiple stimuli at one time. Child development was also assessed using the Peabody Picture Vocabulary Test-4 (PPVT), which provides age-based scores for receptive and expressive language^(^^32^^)^. The tests were administered in the same dedicated clinical research unit by a single individual, with expertise in child psychometric testing. During child testing, the parent(s) were in an adjacent room with a one-way mirror allowing the parent(s) to see the child. All tests were done on the same day, typically taking 90 min with breaks as needed.

### Statistical analysis

All data were analysed using SPSS software (version 15.0; SPSS Inc.). Before statistical analyses, all data were checked for normal distributions using the Shapiro–Wilk test, then mean values and standard deviations or medians, 25th–75th and 2·5th–97·5th percentile ranges calculated, as appropriate. Data showing non-normal distributions were log-transformed for further analyses. Dietary intakes estimated by different methods were compared using ANOVA with Bonferroni correction for multiple comparisons. Potential relationships between the dietary intake of lutein and plasma lutein, and between plasma lutein and the intake of total fat and SFA were examined using Pearson's correlation analysis. The association between child performance on the cognitive tests, plasma lutein and lutein intake was assessed by calculating partial Pearson's correlation coefficients adjusted for potential confounding variables found to be associated with child cognitive development. All variables collected in the demographic data including child sex, breast-feeding duration, ethnicity, maternal IQ, BMI-for-age *Z*-score, number of siblings and number of adults in the household were screened for potential associations. Term gestation at birth and child age were controlled by the study inclusion criteria and not included in these analyses. A *P* value ≤0·05 was considered significant.

## Results

The mean age of the 160 children was 68·7 (sd 0·68) months (range 67–71 months), with 47·5 % boys and 72·5 % of the children of Caucasian background (Table 1). This group of children was primarily from households with two adults, 67 % of the parents reported that they had post-secondary education and <14 % had an income below current definitions for low income in the Province of British Columbia. As required by the protocol, all of the children were born after term gestation, and 89·4 % of the mothers reported that they had breast-fed the child to at least 3 months of age. The mean weight-for-age, height-for-age and BMI-for-age *Z*-scores of the children were 0·18 (sd 0·99), 0·14 (sd 0·97) and 0·10 (sd 1·03), respectively. Also, 5 and 1·25 % of the children had a BMI *Z*-score greater than +2 sd or +3 sd, respectively. No child had BMI, height-for-age or weight-for-age *Z*-score less than –2 sd, respectively, based on the WHO reference data for children.

**Table 1.** Plasma lutein in young Canadian children (Mean values and standard deviations, and medians and percentile ranges)

	Plasma lutein (μmol/l)						
	*n*	%	Mean	sd	Median	25th–75th percentiles	2·5th–97·5th percentiles
All children	160		0·26	0·11	0·23	0·18–0·32	0·11–0·53
Boys	76	47·5	0·27	0·11	0·23	0·19–0·34	0·12–0·54
Girls	84	52·5	0·25	0·11	0·23	0·17–0·32	0·09–0·52
Ethnicity							
Caucasian	116	72·5	0·26	0·11	0·23	0·18–0·32	0·10–0·50
Chinese	19	11·9	0·31*	0·10	0·31	0·21–0·39	0·13–0·51
Other	24	15·0	0·23	0·12	0·20	0·13–0·30	0·11–0·46
Siblings							
Single child	30	18·8	0·30	0·13	0·28	0·21–0·36	0·11–0·59
One sibling	104	65·0	0·25	0·11	0·23	0·17–0·34	0·10–0·46
Two+ siblings	25	15·6	0·23	0·07	0·21	0·20–0·24	0·16–0·47

* Mean value was significantly higher than those of children of other backgrounds (*P* < 0·01).

### Plasma lutein

The distribution of plasma lutein was skewed, with a mean of 0·26 (sd 0·11) μmol/l and median of 0·23 (2·5th–97·5th percentile range 0·11–0·53) μmol/l (Table 1). Plasma lutein concentrations were not different between boys and girls (*P* = 0·43), but were higher in children of Chinese than of other backgrounds (*P* = 0·01), but not Caucasian children (*P* = 0·09). Plasma lutein was also higher in children from families with one rather than two or more adults (*P* = 0·04). We found no significant association between the children's plasma lutein and maternal education (*P* = 0·20), household income (*P* = 0·73), number of children in the family (*P* = 0·10), maternal age (*P* = 0·18), maternal IQ (*P* = 0·59*)*, or child *Z*-scores for weight-for-age (*P* = 0·89), height-for-age (*P* = 0·78) or BMI-for-age (*P* = 0·67).

### Relationship of lutein and macronutrient intake with lutein status

Of the 160 children for whom plasma lutein was analysed, dietary data collected by FFQ were complete for 158 children; 152 children had complete 24 h dietary recall records for the 24 h before blood collection, and 149 had three complete 24 h dietary recall records (Table 2). Fat, protein and carbohydrate provided a mean of 32–33, 16 and 53–54 % daily energy, respectively. SFA, MUFA, *n*-6 and *n*-3 fatty acids provided mean intakes of 12·4–12·5, 11·4–11·7, 4·58–5·03 and 0·61–0·67 % of dietary energy estimated using the three dietary approaches, with no differences among the different dietary approaches (*P* > 0·05; Table 3). Lutein intakes, but not the intakes of energy, protein, fat or carbohydrate, were skewed (*P* < 0·05), with higher mean than median intakes regardless of whether assessed by FFQ, one or three 24 h records (*P* < 0·001; Table 2). More importantly, the median lutein intake was 72–122 % higher when estimated using the FFQ than one or three 24 h records (Table 2). When adjusted for energy intake, lutein intakes remained skewed, with a median intake of 851 µg/4184 kJ (851 µg/1000 kcal), almost twofold higher than the median intakes of 473 and 465 µg/4184 kJ estimated using one or three 24 h records, respectively. Energy, fat, protein and carbohydrate intakes were also higher when assessed using the FFQ than 24 h dietary records (*P* < 0·001); however, overestimation was substantially lower (18–19 % for energy, 14–18 % for fat, 23 % for protein and 21 % for carbohydrate) than for lutein. There were no significant relationships between lutein intake and any child or family characteristic recorded (*P* > 0·05), except that lutein intakes showed a trend to be higher among children with no siblings than children with one or more than one siblings, with medians of 799 (5th–95th percentile range 259–4897, *n* 28), 697 (5th–95th percentile range 264–2942, *n* 97) and 593 (5th–95th percentile range 173–1259, *n* 24) µg/d, respectively (*P* = 0·08).

**Table 2.** Energy, fat and lutein intakes of children assessed using FFQ and 24 h recalls* (Mean values and standard deviations, and medians and percentile ranges)

Intake		Mean	sd	Median	25th–75th percentiles	2·5th–97·5th percentiles
Energy (kJ/d)†	FFQ	7657^a^	2058	7410	6180–9033	4326–12690
	1 × 24 h	6468^b^	1870	6305	5029–7971	3531–10724
	3 × 24 h	6393^b^	1385	6309	5406–7180	3887–9234
Total fat (g/d)	FFQ	66·6^a^	22·3	61·5	50·4–79·4	35·5–118
	1 × 24 h	58·4^b^	24·8	56·4	37·8–73·5	17·4–110
	3 × 24 h	56·4^b^	16·5	56·4	43·5–65·4	26·7–92·2
Lutein (µg/d)	FFQ	2130	2125	1559^a^	958–2463	323–8561
	1 × 24 h	1230	2020	658^b^	389–1219	103–6236
	3 × 24 h	1011	1122	697^b^	475–1064	178–5287
Lutein (µg/4184 kJ)†	FFQ	1188	1132	851^a^	555–1327	189–4585
	1 × 24 h	808	1210	473^b^	270–842	88·6–5097
	3 × 24 h	673	706	465^b^	325–721	138–3264

^a,b^ Values within a column for a given nutrient with unlike superscript letters were significantly different (*P* < 0·05; ANOVA).

* Intakes were determined by FFQ for the previous month for *n* 158, for the one 24 h preceding blood collection for *n* 152, and as the average of three 24 h recalls over 14 d for *n* 149. Lutein, but not energy or fat, intakes were skewed (*P* < 0·05).

† Conversion factor 1 kcal = 4·184 kJ.

**Table 3.** Dietary fatty acid composition (% energy) in young children* (Mean values and standard deviations)

	FFQ (*n* 160)		1 × 24 h (*n* 152)		3 × 24 h (*n* 151)	
	Mean	sd	Mean	sd	Mean	sd
SFA	12·4	2·36	12·5	4·26	12·5	2·69
MUFA	11·4	1·87	11·7	4·02	11·6	2·45
*n*-6	4·58	1·17	5·03	2·53	4·81	1·59
*n*-3	0·61	0·18	0·67	0·44	0·64	0·31

* Intake distributions were skewed for *n*-3 fatty acids (*P* < 0·05), with median intakes of 0·57 (5th–95th percentile range 0·38–1·01), 0·54 (5th–95th percentile range 0·23–1·50) and 0·58 (5th–95th percentile range 0·32–1·20) % energy for the FFQ, one and three 24 h diet records, respectively. No statistically significant differences in the distribution of fatty acid intakes were found using different dietary methods (*P* > 0·05; ANOVA).

Plasma lutein was significantly and positively related to lutein intake when analysed using log-transformed data, with the strongest relationship found when intake was assessed as the average of three 24 h records rather than the 24 h before blood collection or by FFQ (*r* 0·479, *P* < 0·001; *r* 0·242, *P* = 0·003; *r* 0·316, *P* < 0·001, respectively) (Table 4). Results of analysis of the impact of dietary fat and fat quality on plasma lutein status are described for data derived from the three 24 h diet records, with no difference in results based on the FFQ. We found no evidence of any relationship between fat intake (g fat/d, *P* = 0·91; percentage dietary energy from fat, *P* = 0·91) and plasma lutein, although plasma lutein was inversely associated with the percentage dietary fat from saturated fat (*r* –0·205; *P* = 0·01), and positively associated with the percentage fat from *n*-6 fatty acids (*r* 0·176; *P* = 0·032), but not MUFA (*r* 0·042; *P* = 0·609) or *n*-3 fatty acids (*r* 0·062; *P* = 0·454). Children in the highest quartile of SFA intake consumed significantly less lutein, and had lower plasma lutein concentrations than children in the lowest quartile of saturated fat intake, both as a percentage dietary energy or percentage fat intake (data not shown). Therefore, we used stepwise regression to determine the effect of saturated fat intake on plasma lutein, while controlling for lutein intake. This analysis failed to find any association between SFA intake and plasma lutein (*P* = 0·281), indicating that the inverse association between plasma lutein and SFA intake was attributed to differences in lutein intake. Similarly, no association was found between *n*-6 fatty acid intake and plasma lutein (*P* = 0·190).

**Table 4.** Associations between plasma lutein and lutein intake in young children

	Dietary assessment					
	FFQ (*n* 158)		One 24 h recall (*n* 152)		Three 24 h recalls (*n* 149)	
Lutein intake	*r*	*P*	*r*	*P*	*r*	*P*
µg/d	0·316	<0·001	0·242	0·003	0·479	<0·001
µg/4184 kJ†	0·318	<0·001	0·238	0·003	0·490	<0·001

* Results are Pearson correlation coefficients for the association between lutein intake assessed using FFQ, one 24 h recall or as the average of three 24 h recalls.

† Conversion factor 1 kcal = 4·184 kJ.

### Lutein and child cognitive performance

In unadjusted analysis, plasma lutein showed no association with child cognitive test results for the PPVT (*r* 0·131; *P* = 0·104), the KABC-II sequential process (*r* 0·094; *P* = 0·241), simultaneous processing (*r* 0·065; *P* = 0·416), mental processing (*r* 0·075; *P* = 0·360) or learning ability (*r* 0·004; *P* = 0·963). With respect to breast-feeding, our analysis found that children breast-fed >3 months had significantly higher test scores on the PPVT (*P* = 0·037) and the simultaneous processing scale (*P* = 0·034) than children breast-fed <3 months of age. Adjusting for variables associated with child cognitive test scores, including breast-feeding duration, number of adults in the household and ethnicity, did not change this result. Analysis using only the subset of children who had been breast-fed to at least 3 months of age (*n* 143) similarly found no significant association between plasma lutein and cognitive test scores for this group of children. However, although not significant, a trend was evident comparing test scores for children in the lowest compared with the highest quartile of plasma lutein for some tests (Table 5). In addition, lutein intake assessed by any method showed no association with any child cognitive test score.

**Table 5.** Cognitive test scores of children grouped by quartile of plasma lutein* (Mean values and standard deviations)

	Quartile of plasma lutein						
	1 (*n* 40)		2 + 3 (*n* 80)		4 (*n* 40)		
	Mean	sd	Mean	sd	Mean	sd	*P*
Plasma lutein (µmol/l)	80·7	15·1	135	23·4	237	42·8	
PPVT	114	18·3	117	18·4	120	18·0	0·319
KABC-II							
Mental process	76·9	10·7	78·1	11·3	79·7	11·6	0·556
Sequential process	21·3	4·91	21·6	4·58	22·3	3·48	0·593
Simultaneous process	33·7	5·81	34·8	5·16	34·8	6·19	0·580
Learning ability	21·8	4·56	21·8	5·13	22·5	5·00	0·765

PPVT, Peabody Picture Vocabulary Test; KABC-II, Kaufman Assessment Battery.

* No statistically significant differences were found in test performance between children grouped by quartile of plasma lutein concentration (*P* > 0·05; ANOVA).

## Discussion

While information on lutein intakes among adults is growing, relatively little is known about lutein intakes and status, or their significance in young children. In the present report, we show a median plasma lutein of 0·23 µmol/l with a 2·5th–97·5th percentile range of 0·11–0·53 µmol/l among healthy children 5·75 years of age, at low risk for neurodevelopmental delay. Lutein status was best correlated with intake when intake was assessed as the average of three 24 h records collected over 14 d, rather than by FFQ or a single 24 h record. We also show that dietary fat quality is associated with lutein intake in free-living children, although we could find no evidence that dietary fat intake or fat quality had an independent effect on plasma lutein over the range of fat intakes in the present study.

Although 24 h recalls are practical and useful for comparing dietary intakes between large groups, this approach may have limited utility for studies that aim to link the intake of nutrients or foods not eaten daily to nutrient status and health outcomes within individuals rather than among groups. Many pigmented vegetables and fruits, eggs and fatty fish may fall in this category, and when not eaten daily, estimation of intake based on one or a limited number of days may over- or underestimate true average intakes for the individual, limiting the ability to detect important effects of foods or nutrients on health outcomes. As is well known, FFQ are limited by problems of overestimation and desirability bias whereby over-reporting foods is more likely for foods considered healthy. The results of the present study are consistent with and reinforce these problems for the assessment of lutein intake. Lutein intakes of children 5·75 years of age in the present study were almost two-fold higher when assessed using an FFQ than as the average of three or one 24 h recall record, with medians of 851 (2·5th–97·5th percentile range 189–4585), 465 (2·5th–97·5th percentile range 138–3264) and 473 (2·5th–97·5th percentile range 88·6–5097) µg/4184 kJ, respectively. The extent of over-reporting of protein, carbohydrate and fat on the FFQ was considerably lower, suggesting greater bias of parents in overestimating their child's intake of vegetables and fruits as compared with other foods.

Information on lutein intake has been reported from national data and some small studies in other countries. Analyses of a single 24 h recall collected in the US National Health and Nutrition Examination Survey (NHANES) in 2003–2004 estimated lutein intakes among children 4–8 years of age of 311 (sd 474) µg/d, with intakes of 335 (sd 730) and 432 (sd 1062) µg/d for children 9–13 years of age and youth 14–18 years of age^(^^33^^)^. The sd values exceeding the mean indicate wide variation, and skewing. For comparison, the mean lutein intake based on one or three 24 h recall records in the present study were 1230 (sd 2020) and 1011 (sd 1122) µg/d, which may suggest higher intakes of lutein among children in the present study, or possible increased awareness and availability and hence intake of lutein-rich foods over the decade since the 2003–2004 NHANES survey. Regardless, the present results show that 70–75 % of children had lutein intakes below the group mean, with median intakes of 658 (75th percentile 1219) and 697 (75th percentile 1064) µg/d based on the one and three 24 h recall records, which is important for understanding the proportion of the group at risk of low intake.

Regardless of the apparent differences in lutein intake, the median of plasma lutein of 0·30 (5th–95th percentile range 0·18–0·57) µmol/l in US children aged 6–7 years (*n* 839)^(^^34^^)^ is similar to the present results for Canadian children of 0·23 (5th–95th percentile range 0·12–0·48) µmol/l (*n* 160). Similarly, the mean plasma lutein of 0·26 (sd 0·11) µmol/l (*n* 160) for children aged 5·75 years in the present study is similar to concentrations of 0·22 (sd 0·13) µmol/l in US girls (*n* 15) and 0·36 (sd 0·18) µmol/l in boys (*n* 14) aged 4–8 years^(^^22^^)^ and 0·33 (sd 0·16) µmol/l in Australian children (*n* 125) aged 5–12 years^(^^23^^)^. Notably, dietary intakes of lutein estimated as the average of two 24 h recalls for the latter US girls and boys was 345 (sd 491) µg/d^(^^22^^)^, and 2446 (sd 1154) µg/d among the Australian children estimated using an FFQ^(^^23^^)^, are also consistent with the considerable discrepancy in lutein intake estimated using an FFQ compared with dietary recall in the present study (Table 2). Although all plasma lutein must originate from the diet, previous studies on the strength of the correlation between lutein intake and plasma lutein range from no significant association^(^^22^^,^^23^^)^ to correlations ranging from 0·1 to 0·34^(^^17^^–^^20^^)^ to as high as *r* 0·76 (*P* < 0·0001)^(^^21^^)^. Using log-transformed data for dietary lutein intake derived from the average of three 24 h records collected over a 14 d period, we show that differences in lutein intake as µg/d or µg/4184 kJ accounted for almost 25 % of the variability in plasma lutein among the children (*r* 0·479, *P* < 0·001; *r* 0·490, *P* < 0·001, respectively). Correlation coefficients calculated using lutein intakes derived from single 24 h recall records were about 50 % lower (*r* 0·242; *P* = 0·003), probably due to reduced precision of estimating individual average intake using a single day of intake. The FFQ, which averaged intake over the previous month, similarly gave lower, although statistically significant, correlation coefficients (*r* 0·316 and *r* 0·318, respectively, for lutein intake as µg/d or µg/4184 kJ). Intervention studies have shown that plasma lutein increases within days of increased intake of lutein-rich fruits and vegetables^(^^24^^)^, which together with over-reporting may reduce the value of FFQ in addressing the contribution of diet to variability in plasma lutein. In this regard, the lipophilic water-insoluble nature of xanthophylls suggests that fat intake, by enhancing lutein absorption, may influence lutein status. Recent studies raising this question reported that lutein absorption was higher in rodents fed spinach with butter rather than fish oil, and that *in vitro* bioaccessibility of xanthophylls was higher when combined with butter or palm oil rather than olive or fish oil^(^^27^^)^. In the present study, we found no association between fat intake and lutein intake, but children consuming diets richer in SFA consumed less lutein than children consuming more *n*-6 and *n*-3 PUFA. Since dairy foods, processed foods and bakery foods, all of which contain little or no lutein, are major sources of SFA among children in our population^(^^35^^)^, this is not unexpected. After controlling for differences in lutein intake, we found no evidence that dietary fat quality makes an impact on lutein status.

Knowledge that lutein is selectively accumulated in the brain^(^^2^^)^ has raised interest in the possibility that lutein may have important roles in neuroprotection or neural function. In this regard, recent studies have reported that lutein supplementation improved verbal fluency, memory and learning in older adults^(^^11^^)^, and both serum and brain lutein levels were associated with better cognition in octogenarians^(^^12^^)^. Since brain development continues throughout early childhood with an increase in the size of the human brain from about 370 g at birth to 1250 g at 5–6 years of age, and peak synaptic density not being achieved until 5–6 years of age^(^^14^^,^^15^^)^, we also addressed whether lutein status is associated with cognition among the children in the present study. As reported by others^(^^36^^,^^37^^)^, breast-feeding longer than 3 months was associated with better cognitive test scores, with an association between cognitive test performance and number of adults in the household and ethnic background also found in the present study. After adjusting for potential confounding, we found no evidence of an association between plasma lutein and cognitive test scores using the KABC-II and PPVT. However, if breast-feeding or family variables associated with child cognitive test scores are also associated with child diet, which is reasonable, then we may have removed important effects of lutein in child cognition. We also note that because lutein intake is associated with other aspects of diet quality, including SFA intake, it is possible that differences in other nutrients important in neural development are present and also mask important independent effects of lutein.

In summary, the present study has described lutein intakes and status in a relatively large group of young Canadian children at low risk for developmental delay or nutrient inadequacy due to food insecurity. We note that the lutein intake and status of children in the present study may not be representative of children in other parts of Canada, or those who did not participate in the study. The present study highlights the importance of dietary methodology for collecting information on lutein intake for children, showing that three separate days provide better estimates of usual intake with stronger correlations to plasma lutein than single 24 h records or FFQ. Children with higher intakes of saturated fat are also those most likely to have low intakes of lutein-rich foods, but over the range of usual intakes neither fat not fat quality contributes to variations in plasma lutein status. Finally, although we found no evidence that lutein is associated with cognition in healthy young children at low risk for poor outcome, it is possible that lutein plays an important role in the brain related to neuroprotection, contributing to preservation of function in ageing, neurological disease or high-risk newborns. Alternatively or in addition, lutein may exert subtle independent effects on child cognition that are difficult to separate from covariates that make an impact on both dietary patterns and child cognition.
